# The relationship between teachers’ disciplinary practices and school bullying and students’ satisfaction with school: The moderated mediation effects of sex and school belonging

**DOI:** 10.1371/journal.pone.0303466

**Published:** 2024-05-28

**Authors:** Marina Kovacevic Lepojevic, Marija Trajkovic, Luka Mijatovic, Branislava Popovic-Citic, Lidija Bukvic, Milica Kovacevic, Ana Parausic Marinkovic, Mladen Radulovic

**Affiliations:** 1 Institute for Educational Research, Belgrade, Serbia; 2 University of Belgrade, Faculty of Special Education and Rehabilitation, Belgrade, Serbia; 3 Institute of Criminological and Sociological Research, Belgrade, Serbia; University of Study of Bari Aldo Moro, ITALY

## Abstract

An authoritative school climate, along with greater teacher support and warm relations among peers are frequently connected with less school bullying. The main aim of this paper is to examine the direct link as perceived by students between teachers’ disciplinary practices and bullying in school and students’ satisfaction with school. The indirect relationships are explored via the mediation of school belonging and the moderation of sex. High school students (N = 860, 40.4% male students) completed the Delaware School Climate Survey, the Multidimensional Students’ Life Satisfaction Scale, and the Psychological Sense of School Membership Scale at a single time point. In general, teachers’ disciplinary practices have significant direct effects on perceptions of bullying and satisfaction with school. Positive disciplinary (direct effect = .28, SE = .04) and SEL techniques (direct effect = .22, SE = .04) are related to bullying only among males, while punitive techniques are directly linked to school bullying unrelated to sex (b = .03, SE = .05). Similarly, the effect of positive disciplinary (direct effect = .27, SE = .08) and SEL (direct effect = .21, SE = .08) techniques on satisfaction with school was significant only among males. A direct relationship between punitive disciplinary techniques and satisfaction with school was not recognized. The mediation analysis revealed the indirect effects of teachers’ disciplinary practices on the dependent variables via school belonging to be stronger among females. Teachers’ negative modeling through punitive disciplinary practices leads to more bullying. School belonging may serve as a protective factor related to the negative impact of teachers’ disciplinary practices on school bullying as well as satisfaction with school, especially among females. Interventions should be focused on fostering school belonging along with the development of positive sex-specific disciplinary practices.

## Introduction

By its definition school bullying involves the repeated intent to harm and an imbalance of power between the aggressor and the victim [1]. Such an imbalance of power may stem from physical strength, social status within the group, or a certain vulnerability (e.g. appearance, learning difficulties, family situation, personality characteristics) [2]. Less school bullying is frequently connected with an authoritative school climate, and more teacher, peer and parental support [3–5]. Scientific results imply that instead of being considered in terms of the individual’s behaviour, bullying should be considered as a structural issue [6]. Harsh discipline in schools is generally directly related to more experiences of bullying as a consequence of negative teacher-student modeling [7]. Punishment is often used in traditionally oriented schools and reflects a policy of zero tolerance and the frequent use of suspensions and exclusions from school. Research results indicate that a supportive disciplinary framework is recognized in effective bullying prevention programmes [8], and even punitive discipline may be successful in achieving the short-term effects of managing student behaviour [9]. Teachers’ SEL disciplinary practices are the most effective in developing students’ self-discipline and long-term positive developmental changes [10]. The results of evaluation studies show that SEL in combination with positive disciplinary techniques achieves better results than without them [11, 12]. Certain authors stress that in an authoritative school climate, both responsiveness (support) and demandingness (structure) are equally valued, and together are viewed as instrumental for effective discipline in both the short and long term [13]. There has been a notable shift in school programmes from bullying prevention to the systemic integration of the evidence-based practices of social and emotional learning (SEL) [14]

One of the frequently examined indicators of positive youth developmental outcomes is student life satisfaction [15]. Subjective well-being is most often interpreted as experiencing a high level of positive affect, a low level of negative affect, and a high degree of satisfaction with one’s life [16]. The concept of subjective well-being has frequently been used synonymously with ‘‘happiness”, meaning that maximising one’s well-being has been viewed as maximising one’s feelings of happiness [16]. However, self- reports of being happy do not necessarily mean that people are psychologically well [16]. As represented in the Eudaimonic Activity Model, eudaimonic and hedonic aspects of well-being are closely related [17]. Life satisfaction is one of the most important indicators of youth well-being and represents their cognitive evaluation of their quality of life [18]). This might be conceptualized as a general life satisfaction assessment or within specific life domains (e.g. satisfaction with friends, family, and school experiences) [19, 20]. The author suggests the variability in satisfaction ratings across life domains, with adolescents reporting the greatest dissatisfaction with their school experiences [21]. Creating a balance between responsiveness and demandingness in the classroom is connected to higher student satisfaction with school [22]. The interpersonal relations between students and teachers and among peers has been found to be the most important school climate factor which affects student satisfaction with school [22]. The research results suggest that teachers should focus more on positive disciplinary practices as they are linked to improved outcomes for both students and teachers [23]. Monitoring the effects of the RULER program–an evidence-based approach to social and emotional learning, significant improvements in multiple dimensions of the school climate, including disciplinary practice, were found to be related to satisfaction with school [24].

Previous research recognized school belonging as a good mediator in explaining the link between different aspects of the school climate and various positive and negative student outcomes such as problematic internet use [25]; academic success [26]; bullying, and symptoms of depression [27], etc. School belonging is defined as the extent to which students feel personally accepted, respected, included, and supported by others in the school social environment [28]. The research results indicate that school belonging is closely related to many positive developmental outcomes such as higher student cognitive and behavioural engagement, higher motivation and academic success [29, 30]; fewer problems and higher prosocial behaviour [31, 32], and higher life satisfaction [33]. The results show that students in positive school climates report higher levels of school belonging and fewer physical, emotional, and cyberbullying behaviours [27, 34]. School belonging mediates certain school climate aspects (e.g. teacher-student relationships, and a sense of fairness) in relation to students’ life satisfaction [35]. School belonging may not be relevant for negative disciplinary practices. Supportive teaching practices are closely linked to school connectedness, while punitive disciplinary practice has no significant correlation with school connectedness [36]. Class level path analysis showed that the effect of the student-teacher relationship on bullying behaviour is mediated by the student-student relationship [37].

Numerous differences in peer socialization and variances between males and females have been recognized to date, starting from girls’ relational orientation, a tendency to build more meaningful relationships, interpersonal sensitivity and prosocial orientation [38], suggesting greater male engagement in bullying [5, 39], less connectedness with school [40], and less positive relationships with teachers [41, 42]. To our knowledge, sex moderation of the mediation of school belonging in examining the relationship between teacher disciplinary practices and school bullying behaviour and satisfaction with school has not yet been explored.

**Fig 1:**
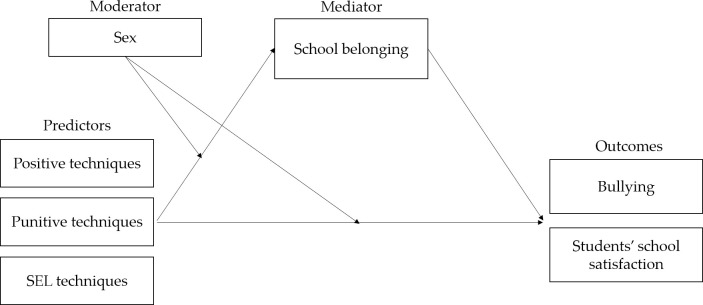
Conceptual model for moderated mediation.

The main aim of this paper is to examine the links between teachers’ disciplinary practices perceived by students with bullying in school and with students’ satisfaction with school. The indirect relationships are explored via the mediation of school belonging and the moderating role of sex. The proposed model includes how (the mediating effect), when (the moderating effect), and when and how (moderated mediating effect) teachers’ disciplinary practices affect both bullying and students’ school satisfaction (Fig 1).

Based on authoritative discipline theory [43, 44] and Stockard and Mayberry’s [45] theoretical framework for the school climate which imply that a healthy balance between responsiveness (support) and demandingness (structure) lead to more self-discipline [46] we hypothesized that: punitive discipline is negatively related to satisfaction with school and positively to bullying; teachers’ socio-emotional techniques as well positive disciplinary techniques are negatively related to school bullying and positively to students’ satisfaction with school. School belonging is expected to have mediating potential in the explanation of the relationship between disciplinary practices and bullying in schools and disciplinary practices and students’ satisfaction with school. Additionally, we expect to find that the proposed interactions differ by sex We hypothesized that socioemotional learning and positive disciplinary techniques can help to develop school belonging, especially at females. Also, we expect that teachers’ negative modeling via the use of punitive discipline is expected to affect males more directly. We consider that further exploring of the sex moderation between teacher practices and school belonging can help to the developing of school belonging, greater satisfaction of students and prevention of school bullying.

## Materials and methods

Students from 11 Belgrade (Serbia) high schools from the first to the fourth grade (N = 860, 40.4% male students) completed the Delaware School Climate Survey [47], the Multidimensional Students’ Life Satisfaction Scale [19], and the Psychological Sense of School Membership Scale [28] at a single time point from April 5^th^ to May 28^th^ 2021. *Measures* The Delaware Positive, Punitive, and SEL Techniques Scale [47] measures students’ perceptions of the extent to which three types of techniques are used in their school to manage student behaviour. The positive behaviour techniques consist of 4 items (e.g. Students are often praised), the use of punitive/corrective techniques of 4 items (e.g. Students are punished a lot), and the use of SEL techniques of 5 items (e.g. Students are taught to feel responsible for their behaviour). In the current study, Cronbach’s alpha coefficient ranged from 0.77 (Punitive Techniques), 0.83 (Positive Techniques) to 0.85 (SEL Techniques) and McDonald’s omega from 0.77 (Punitive Techniques), 0.84 (Positive Techniques) to 0.85 (SEL Techniques) for the student sample. The Bullying School-Wide subscale comprises 4 items which explore students’ perceptions of bullying in their schools (e.g. In this school, bullying is a problem). The rating response range was from 1 (strongly disagree) to 4 (strongly agree). The internal consistency measured by Cronbach’s alpha and McDonald’s omega coefficient is 0.76. The Multidimensional Students’ Life Satisfaction Scale (MSLSS) [19] is a 6-point Likert-type self-report scale (ranging from strongly disagree (1) to strongly agree (6)), designed for children aged 8 to 18. The MSLSS is designed to provide a holistic assessment of the wellbeing of young people. It has five subscales: family, friends, school, the living environment and self. The school domain items rep-resent satisfaction with school life (e.g. I enjoy school activities). The value of Cronbach’s alpha is 0.84 and McDonald’s omega is 0.85. The Psychological Sense of School Membership Scale (PSSM) comprises 18 items (e.g. Most teachers at this school are interested in me.) to be assessed on a 5-point Likert-type scale from 1 (strongly disagree) to 5 (strongly agree) [28]. The internal consistency is 0.84 measured by Cronbach’s alpha and 0.90 measured by McDonald’s omega.

*Procedure*. Verbal informed consent from participants is obtained. At first we presented the research and relevant procedures involve with data collection and usage, and ask to declare if someone doesn’t want to participate. It was witnessed by school psychologist. One school hour was necessary for completing the questionnaire. In the Republic of Serbia there is no regulation at all about parental consent for children participating in scientific research, but Family law, Official Gazette, no 18/2005, 72/11 and 6/2015,and Law about protection of the rights of the patients, Official Gazette, no 45/2013-19, 25/2019-3, respect privacy of children above 15 years (e.g. they make decisions about medical treatments by their own). This study was approved by the Research Ethics and Conduct Committee of the CEPORA–Center for Positive Youth Development (no. 12/2021 ES)

## Results

### Descriptive statistics and the intercorrelations of the variables

Descriptive and correlation analyses were conducted using SPSS 21.0. PROCESS analyses were performed to test the mediating role of school belonging in predicting bullying and students’ school satisfaction by teachers’ disciplinary practices (positive, punitive and SEL techniques) along with the moderating role of sex. The entire model was previously tested in IBM AMOS version 25.

Table 1 presents the means and standard deviations of the study variables: positive, punitive and SEL techniques, school belonging, bullying and students’ school satisfaction and their intercorrelations.

**Table 1:** Descriptive and correlation analyses of the study variables.

Variable	N	M	Range	SD	Sk(SE)	Ku(SE)	1	2	3	4	5	6
1 Positive techniques	786	15.4	6–24	5.1	-.08(.09)	-.90(.17)						
2 Punitive techniques	790	13.8	6–24	4.4	.24(.09)	-.69(.17)	-.15**					
3 SEL techniques	786	16.5	6–24	5.1	-.27(.09)	-.76(.17)	.76**	-.09*				
4 School belonging	742	65.7	23–90	14.5	-.46(.09)	-.36(.18)	.53**	-.40**	.58**			
5 Bullying	804	9.2	4–16	3.5	.13(.09)	-.86(.17)	-.08*	.55**	-.14**	-.42**		
6 Students’ school satisfaction	812	29.3	8–48	10.1	-.21(.09)	-.60(.17)	.47**	-.36**	.49**	.76**	-.33**	

Notes.

* p < 0.05

** p < 0.01

The correlation results indicated that bullying was negatively associated with SEL techniques, school belonging and students’ school satisfaction (p < 0.01) and positively with punitive techniques (p < 0.01). Students’ school satisfaction was negatively related to punitive techniques, bullying and age (p < 0.01) and positively to the other study variables. The correlations of age with positive techniques, SEL techniques, school be-longing and students’ school satisfaction were negative and weak (p < 0.01).

### Moderated mediation analyses results

The entire model was previously tested in IBM AMOS version 25. Results suggested the model can be considered unsatisfactory: Chi-square value was significant (χ2 = 780.396, p < .001), the TLI and CFI values were below the recommended threshold of 0.90, while the RMSEA was far above the prescribed value of 0.08 (TLI =.-.111, CFI = .704, RMSEA = .339). For that reason, direct and indirect effects were separately tested using the SPSS macro PROCESS suggested by Hayes [48]. Using proposed model number 59, six PROCESS analyses were conducted–one for each pair of predictor and dependent variables. In this way the independent contribution of the predictors and their relations with the mediator were examined with sex as the moderator. Age was treated as a covariate and its role will not be presented within the results or discussed. A full information maximum likelihood estimator was used which could also handle missing values. The direct, indirect, and total effects were calculated. 5000 bootstrap samples were used. Bootstrapping is a non-parametric method which bypasses the is-sue of non-normality distribution [49]. All used variables (except sex) were standardized prior to test and effects and their standard errors (SE) are shown.

**Fig 2:**
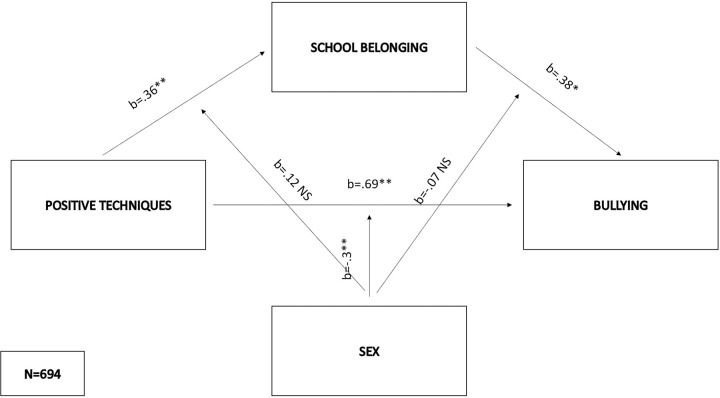
Moderated mediation model with positive techniques as the predictor and bullying as the criterion.

As regards the first model (F(5,688) = 40.86, p < .01), positive techniques positively predicted school belonging (b = .36, SE = .11, p < .01), sex also predicted the mediator variable (b = -.16, SE = .007, p < .05), while the interaction between positive techniques and sex on school belonging was not significant. Bullying was negatively predicted by school belonging (b = -.38, SE = .15, p < .05) and positively by positive techniques (b = .69, SE = .14, p < .01). Interaction of sex and positive techniques negatively predicted bullying (b = -.3, SE = .08, p < .01) which explained 1.5% of the variance in predicting bullying, while interaction of sex and belonging didn’t have significant effect on dependent variable. Further probing of the significant interaction indicated that the conditional direct effect was significant (and positive) only for males (direct effect = .39, SE = .06, p < .01), while the indirect effects of positive techniques on bullying did not differ between males and females–the index of moderated mediation was not significant (index = -.09, bootSE = .052, 95% BootLLCI = -.2—BootULCI = .01). The entire first model explained 22,9.% of the variance in bullying (Fig 2).

**Fig 3:**
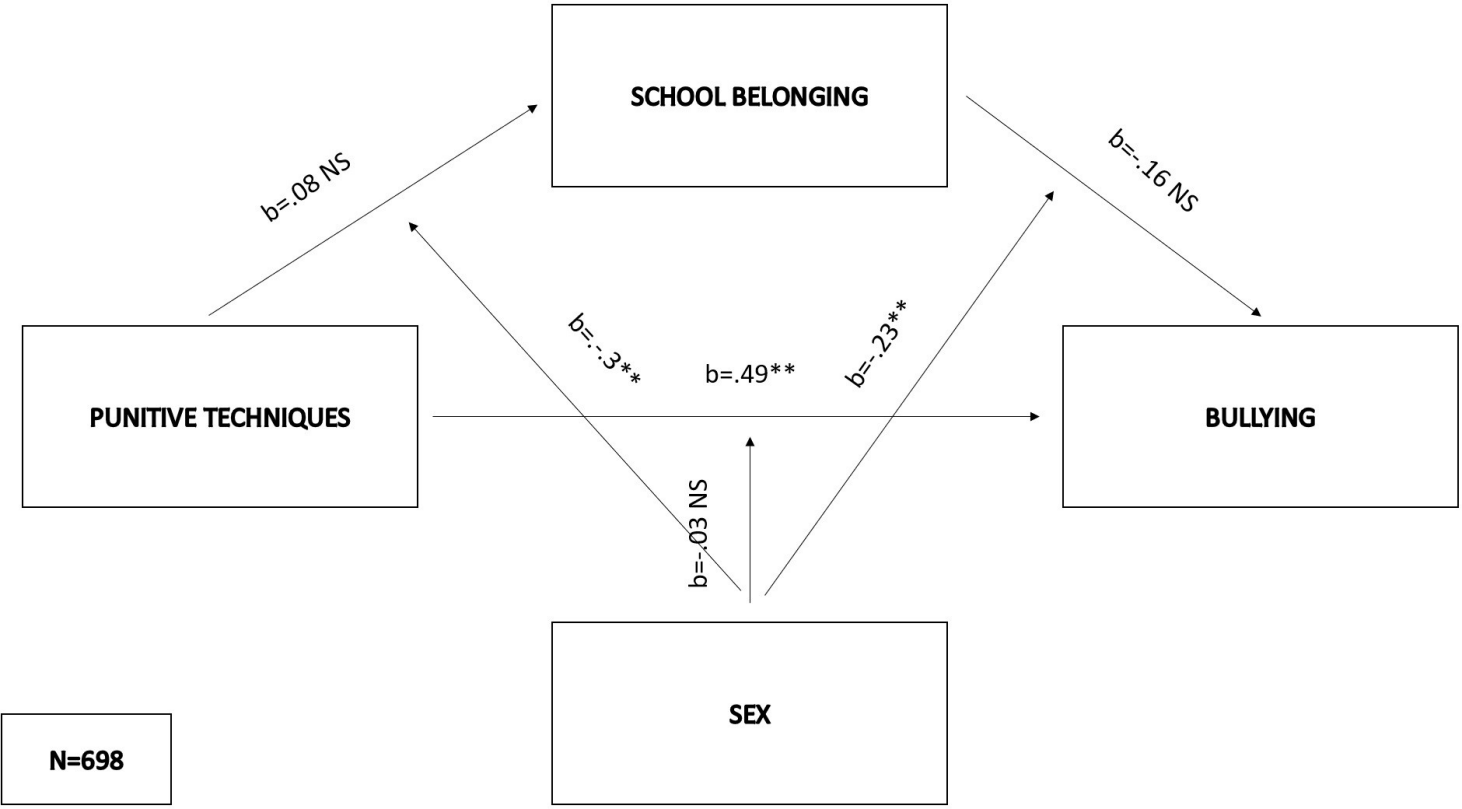
Moderated mediation model with punitive techniques as the predictor and bullying as the criterion.

Within the second model (F(5,692) = 80.09, p < .01), punitive techniques (b = .11, SE = .38, p = .47) and sex (b = -.13, SE = .07, p = .06) as the predictors did not affect school belonging, while the interaction of sex and punitive techniques (b = -.30, SE = .07, p < .01) was significant in predicting the mediator. This interaction explained an additional proportion of the variance in school belonging (2%) with significant effects in both males (effect = -.22, SE = .05, p < .01) and females (effect = -.52, SE = .04, p < .01), showing that punitive techniques exerted a stronger negative effect on school belonging among females. Bullying was positively predicted by punitive techniques (b = .49, SE = .1, p < .01) and sex (b = .22, SE = .06, p < .001), while belonging didn’t have significant effect on bullying in context of these variables (b = .16, SE = .12, p = .18). Interaction of sex and punitive techniques (b = -.03, SE = .06, p = .63) did not influence the criterion, but interaction between belonging and sex did (b = -.23, SE = .07, p < .01), explaining an additional proportion of the variance in bullying (1%) and showing that there is significant negative effect of belonging on bullying among girls (effect = -.31, SE = .04, p < .01) and no such effect among boys. Consequently, the indirect effects of punitive techniques on bullying were positive and significant females (indirect effect = .16, bootSE = .02, 95% BootLLCI = .12—BootULCI = .21) and unsignificant for males (indirect effect = .02, bootSE = .01, 95% BootLLCI = -.01—BootULCI = .04). The index of moderated mediation was significant (index = .14, bootSE = .03, 95% BootLLCI = .09—BootULCI = .20). The entire second model explained 37% of the variance in bullying (Fig 3).

**Fig 4:**
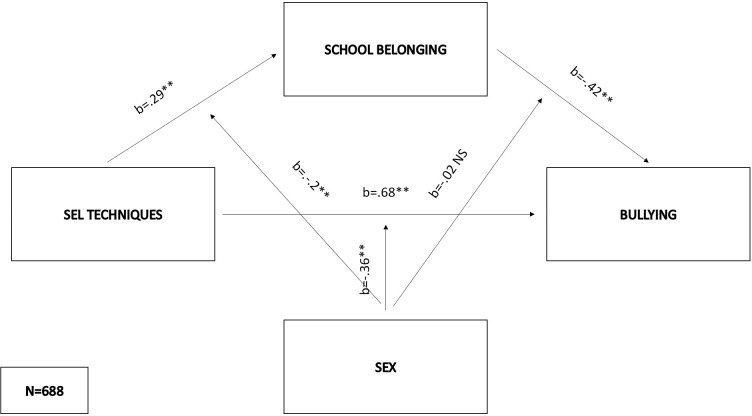
Moderated mediation model with SEL techniques as the predictor and bullying as the criterion.

As regards the third model (F(5,682) = 37.65, p < .01), school belonging was predicted by SEL techniques (b = .29, SE = .1, p < .01), sex (b = -.24, SE = .06, p < .01) and their interaction (b = .2, SE = .06, p < .01). The sex-SEL interaction explained an additional 1% of the variance in the mediator with significant positive effects in both males (effect = .49, SE = .05, p < .01) and females (effect = .69, SE = .04, p < .01), indicating again a stronger effect among females. Bullying was positively predicted by SEL techniques (b = .68, SE = .14, p < .01) and negatively by school belonging (b = -.42, SE = .16, p < .01). Sex (b = .1, SE = .07, p = .15) and the sex x belonging interaction (b = -.02, SE = .09, p = .81) didn’t significantly influence the criterion, while sex x SEL techniques interaction had significant affect (b = -.36, SE = .09, p < .01) This interaction explained 2% of the variance in bullying. The conditional direct effect of SEL techniques on bullying was significant for males (direct effect = .32, SE = .06, p < .01), but not for females. The indirect effects were significant for both males (indirect effect = -.22, bootSE = .04, 95% BootLLCI = -.30—BootULCI = -.13) and females (indirect effect = -.32, bootSE = .04, 95% BootLLCI = -.40—BootULCI = - .25). and they did not significantly differ between males and females (index = -.11, bootSE = .06, 95% BootLLCI = -.22—BootULCI = .01). The entire first model explained 22,93% of the variance in bullying (Fig 4).

**Fig 5:**
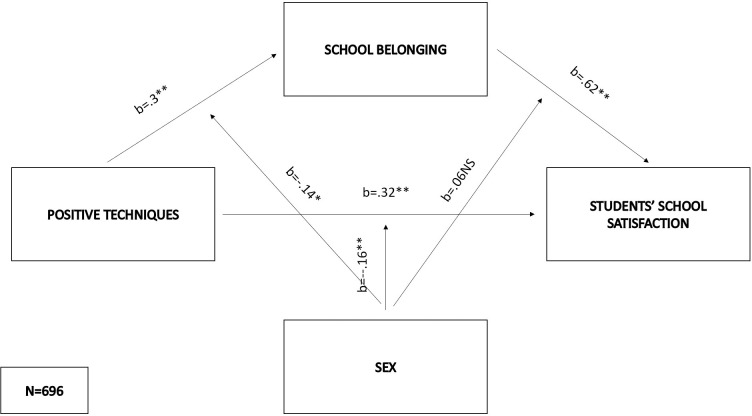
Moderated mediation model with positive techniques as the predictor and students’ school satisfaction as the criterion.

As regards the fourth model (F(5,690) = 192.73, p < .01), the prediction of the mediator was significant for positive techniques (b = .3, SE = .11, p < .01), sex (b = -.19, SE = .06, p < .01) and their interaction (b = .14, SE = .07, p < .05), while the effects on the mediator were significant among both males (effect = .44, SE = .05, p < .01) and females (effect = .58, SE = .04, p < .01). This interaction is significantly higher among females. When it comes to predicting students’ school satisfaction, significant effects were found for positive techniques (b = .32, SE = .1, p < .01), school belonging (b = .62, SE = .12, p < .01), sex (b = .15, SE = .05, p < .01), and the sex x positive techniques interaction (b = -.16, SE = .06, p < .01), while sex x school belonging interaction didn’t predict school satisfaction. The conditional direct effect was significant just for males (direct effect = .16, SE = .05, p < .001). The indirect effects were significant for both males (indirect effect = .30, boot SE = .04, 95% BootLLCI = .23—BootULCI = .38) and females (indirect effect = .43, bootSE = .04, 95% BootLLCI = .36—BootULCI = .5), again indicating a stronger effect among females. The index of moderated mediation was significant (index = .13, bootSE = .05, 95% BootLLCI = .03—BootULCI = .23), while the whole model explained 58% of the variance in students’ school satisfaction (Fig 5).

**Fig 6:**
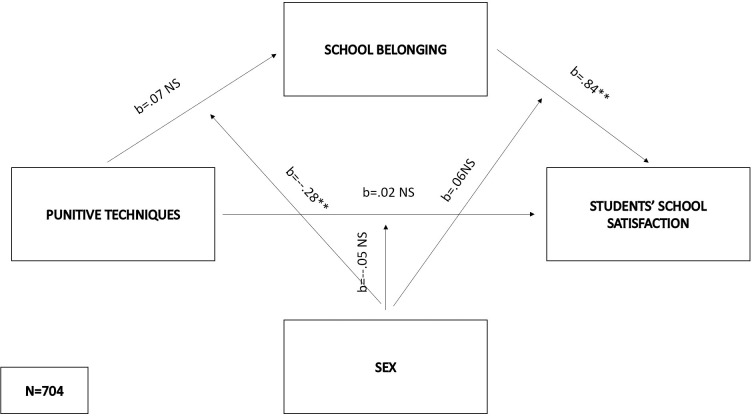
Moderated mediation model with punitive techniques as the predictor and students’ school satisfaction as the criterion.

In the fifth model (F(5,698) = 198.93, p < .01), the mediator variable was predicted by sex (b = -.17, SE = .07, p < .01) and the interaction of sex and punitive techniques (b = -.24, SE = .07, p < .01), which explained 2% of the variance in school belonging. The independent variable alone did not affect the mediator (b = .07, SE = .11, p = .57). The negative effects of the interaction on the mediator were significant in both males (effect = -.22 SE = .05, p < .01) and females (effect = -.5, SE = .04, p < .01), but higher among females. Students’ school satisfaction was directly predicted by school belonging (b = .84, SE = .1, p < .01) and sex (b = .15, SE = .05, p < .001), while the influences of punitive techniques (b = .02, SE = .09, p = .86), and interaction and sex (b = -.05, SE = .05, p = .35) and interaction of school belonging and sex (b = -.06, SE = .06, p = .3) were insignificant. Due to the latter results, the conditional direct effects were not shown. The indirect effects were significantly higher among females (indirect effect = -.36, bootSE = .04, 95% BootLLCI = -.44—BootULCI = -.29) than males (indirect effect = -.17, bootSE = .04, 95% BootLLCI = -.25—BootULCI = -.1). The index of moderated mediation was significant (index = -.19, bootSE = .05, 95% BootLLCI = -.3—BootULCI = -.09), while the model in total explained 59% of the variance in students’ school satisfaction (Fig 6).

**Fig 7:**
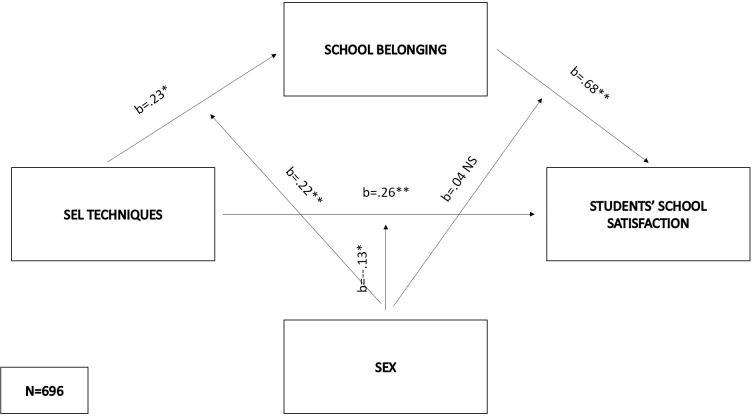
Moderated mediation model with SEL techniques as the predictor and students’ school satisfaction as the criterion.

As regards the sixth model (F(5,690) = 203.05, p < .01), SEL techniques (b = .23, SE = .1, p < .05), sex (b = -.27, SE = .06, p < .01) and their interaction (b = .22, SE = .06, p < .001) predicted the mediator. The SEL techniques x sex interaction explained 1% of the variance of school belonging, while the positive effects on the mediator were significant and lower for males (effect = .45 SE = .05, p < .01) compared to females (effect = .68, SE = .04, p < .01). The effects of SEL techniques (b = .26, SE = .1, p < .01), school belonging (b = .68, SE = .12, p < .001), sex (b = .13, SE = .05, p < .01) and sex x SEL techniques (b = -.13, SE = .06, p < .05) were significant predictors of school satisfaction, while school belonging x sex interaction didn’t have effect. Sex influenced the relation between the independent and criterion variables, where the conditional direct effect was significant only for males (direct effect = .12, SE = .05, p < .01). The indirect effects were significant for both males (indirect effect = .32, bootSE = .04, 95% BootLLCI = .25—BootULCI = .4) and females (indirect effect = .51, bootSE = .04, 95% BootLLCI = .43—BootULCI = .59), with a stronger effect among females once again. The index of moderated mediation was significant (index = .19, bootSE = .05, 95% BootLLCI = .08—BootULCI = .31), while the model in total explained 60% of the variance in students’ school satisfaction (Fig 7).

## Discussion

As expected, punitive discipline is directly and positively related to school bullying unrelated to sex, indicating the impact of teachers’ negative modeling on student-student behaviour and bullying as its negative manifestation [7, 43]. Unexpectedly, positive disciplinary and SEL techniques are positively related to bullying, and that link is found to be relevant only for male students which is contrary to previous findings [24, 50, 51]. One of the explanations for this is that SEL might be perceived as a Trojan horse to increase classroom management and social control, instead of cultivating the positive, full development of the child and the adult educator, including care-givers [31]. The positive relation between positive disciplinary techniques and SEL with school bullying might be explained by difficulties in implementation at secondary school level if such techniques are not applied appropriately at previous educational levels and simultaneously in other ecological contexts. These findings might imply the need to apply systemic SEL as an approach to create equitable learning conditions which actively involve all Pre-K to Grade 12 students in learning and developing social, emotional, and academic competencies [52, 53]. Difficulties in school staff motivation and capabilities are also recognized [54]. Positive and SEL disciplinary techniques which are not properly implemented might be perceived as more teacher- oriented [54]. Teachers are then perceived to have a greater share of the power similar to punitive school discipline. These positive relations might be additionally explained in a reactive way, meaning that teachers respond to severe bullying behaviour inappropriately. Teachers might not recognize when incidents of bullying should be considered as severe, requiring help from other agencies and services. The author has already noticed that the severity of peer victimization may moderate the relationship between socio-emotional learning and school bullying [51]. As the victims of bullying have said, teachers often react to the perpetrator, without offering any support to the victim and the whole class after such incidents [55]. Teachers may underestimate verbal incidents of bullying, even suffer from bullying themselves, or enable bullying by their inappropriate reaction to the bulling incidents which occur in the classroom [56, 57]. As has already been noted in previous research, male students tend to “ignore incidents”, or report only more severe incidents of bullying compared to female students who are more sensitive to minor incidents, which might affect findings about gen-der-specific interactions [58].

The interesting finding that positive discipline and SEL directly relate to student satisfaction with school only for male students might be explained by the fact that although females are more rationally oriented, have positive relations with teachers [41, 42], and build better relations with their peers [58], parents [59] and other important figures, supportive teacher relationships might mean more to male students than their female counterparts. Even punitive disciplinary practice was not directly related to satisfaction with school, while school belonging was found to be a good protective factor especially among female students, which is not surprising because of their relational and contextual orientation [38]. The full mediation of school belonging established in relation to SEL and positive disciplinary techniques on students’ satisfaction with school implies that interventions focused on fostering school belonging along with efforts toward establishing a positive school climate might positively affect students’ wellbeing and have a negative effect on school bullying [24, 32]. According to the current study, school belonging is sex-specific and partly explains bullying behaviour [27, 58] and student wellbeing [34, 35]. The qualitative differences between female and male students indicate that both might use bullying as a tool to feel a sense of belonging, girls to prevent being excluded from the group and boys to avoid being perceived as weak [58].

As has already been noted, bullying does not occur in a vacuum [60]. This study highlights the importance of the disciplinary strategies used by teachers in schools, how they manage their classrooms and how this is related to bullying and satisfaction with school. The data about sex relevance within the examined interactions are of special value in this study. The research results are in line with a noticeable shift in bullying prevention towards evidence-based practices of social and emotional learning (SEL) leading to a variety of positive outcomes for students and teachers alike [14, 50]. Interventions for developing school belonging are highly recommended in order to prevent school bullying and improve students’ positive developmental outcomes. We recommend that a good fit for bullying prevention in middle and high schools might have teacher practice of adopting greater youth participation at classroom level along with practicing SEL. It’s expected that greater youth participation, and more group discussion will strengthen school belonging, equity impact and less bullying [61].

This study is limited by its cross-sectional design. Longitudinal or intervention re-search is necessary to provide more detailed answers to the research question regarding the relation between school climate aspects and school bullying. A further limitation relates to the lack of a class level analysis which could provide more exact data. Longitudinal data could provide some evidence of both the short and long term effects of teachers’ disciplinary practices. This research is mono-informant in nature with the measurements being restricted to student self-rating scales. For future studies we recommend involving more global measures of students’ life satisfaction so as to avoid the similarity between the school belonging and satisfaction with school constructs used in the current study. Additionally, measuring bullying victimization as well as disciplinary infractions would be important in order to gain a better under-standing of the mechanisms underlying teachers’ disciplinary practices.

## Supporting information

S1 Dataset (XLS)
